# *Corynebacterium glutamicum* as an Efficient Omnivorous Microbial Host for the Bioconversion of Lignocellulosic Biomass

**DOI:** 10.3389/fbioe.2022.827386

**Published:** 2022-04-01

**Authors:** Apurv Mhatre, Somnath Shinde, Amit Kumar Jha, Alberto Rodriguez, Zohal Wardak, Abigail Jansen, John M. Gladden, Anthe George, Ryan W. Davis, Arul M. Varman

**Affiliations:** ^1^ Chemical Engineering Program, School for Engineering of Matter, Transport, and Energy, Arizona State University, Tempe, AZ, United States; ^2^ Department of Bioresource and Environmental Security, Sandia National Laboratories, Livermore, CA, United States; ^3^ Department of Biomaterials and Biomanufacturing, Sandia National Laboratories, Livermore, CA, United States; ^4^ Joint BioEnergy Institute, Emeryville, CA, United States

**Keywords:** lignocellulosic biomass hydrolysate, ^13^C-fingerprinting, lignin-derived aromatics, mixed-acid fermentation, L-lactate

## Abstract

*Corynebacterium glutamicum* has been successfully employed for the industrial production of amino acids and other bioproducts, partially due to its native ability to utilize a wide range of carbon substrates. We demonstrated *C. glutamicum* as an efficient microbial host for utilizing diverse carbon substrates present in biomass hydrolysates, such as glucose, arabinose, and xylose, in addition to its natural ability to assimilate lignin-derived aromatics. As a case study to demonstrate its bioproduction capabilities, L-lactate was chosen as the primary fermentation end product along with acetate and succinate. *C. glutamicum* was found to grow well in different aromatics (benzoic acid, cinnamic acid, vanillic acid, and p-coumaric acid) up to a concentration of 40 mM. Besides, ^13^C-fingerprinting confirmed that carbon from aromatics enter the primary metabolism via TCA cycle confirming the presence of β-ketoadipate pathway in *C. glutamicum*. ^13^C-fingerprinting in the presence of both glucose and aromatics also revealed coumarate to be the most preferred aromatic by *C. glutamicum* contributing 74 and 59% of its carbon for the synthesis of glutamate and aspartate respectively. ^13^C-fingerprinting also confirmed the activity of ortho-cleavage pathway, anaplerotic pathway, and cataplerotic pathways. Finally, the engineered *C. glutamicum* strain grew well in biomass hydrolysate containing pentose and hexose sugars and produced L-lactate at a concentration of 47.9 g/L and a yield of 0.639 g/g from sugars with simultaneous utilization of aromatics. Succinate and acetate co-products were produced at concentrations of 8.9 g/L and 3.2 g/L, respectively. Our findings open the door to valorize all the major carbon components of biomass hydrolysate by using *C. glutamicum* as a microbial host for biomanufacturing.

## Introduction

Research efforts are underway worldwide for production of biofuels and chemicals from various renewable resources as a sustainable alternative to fossil resources (Zhou et al., 2008; Zhou et al., 2011; Varman et al., 2013; Salvachua et al., 2015; Liu et al., 2017; Wu et al., 2017). Amongst the various renewable resources that are currently being investigated, lignocellulosic biomass represents the largest net resource with a worldwide annual production of 200 billion tons (Simmons et al., 2008; Tuck et al., 2012). Industrial biotechnology, wherein microbial strains are utilized for coupled bioconversion of various carbon substrates present in biomass, is expected to provide a scalable and cost-competitive production method for renewable fuels and chemicals (Sievert et al., 2017; Flores et al., 2020; Abdel-Rahman et al., 2021; Flores et al., 2021; Wu et al., 2021; Zan et al., 2021). However, several challenges still need to be overcome for efficient bioconversion of biomass to bioproducts. The first challenge is the structural heterogeneity of lignocellulosic biomass, resulting in hydrolysates that contain diverse substrates such as sugars, organic acids and aromatics as the main depolymerized components (Bruijnincx, 2014; Socha et al., 2014). Furthermore, aromatics derived from hydrolysis of lignocellulosic biomass can be inhibitory to the growth of common model microbial conversion hosts such as *Escherichia coli* and *Saccharomyces cerevisiae* (Fitzgerald et al., 2004; Adeboye et al., 2014; Varman et al., 2018). Although significant progress is underway for utilization of each of the potential fermentation substrates, the diversity of these substrates can result in spiralling costs for upstream separations, supplementation of fermentation medium, serial fermentation steps, and product purification. From a techno-economic perspective, bottlenecks associated with any one of these unit operations can tip the scales against economic viability for a promising biotechnology. Process integration provides a means for achieving dramatic improvements in reaction kinetics, yields, product titers, and separations, thereby minimizing cost and risk associated with biomanufacturing (Van Gerven and Stankiewicz, 2009). One-way bioprocess integration could be achieved for valorization of lignocellulosic biomass is by employing omnivorous microbial cell factories that can utilize diverse carbon substrates present in biomass hydrolysates.

*Corynebacterium glutamicum* is a fast growing, aerobic, and non-pathogenic Gram-positive soil bacterium exhibiting physiological traits that could make it an interesting alternative to these model microbes for the reasons discussed below. *C. glutamicum* naturally has a wide substrate range and has been further engineered to uptake other carbon substrates such as xylose and arabinose (Kawaguchi et al., 2008; Xia et al., 2016). Owing to the industrial importance of *C. glutamicum*, the genomes of several of its strains have been sequenced (Ikeda and Nakagawa, 2003; Kalinowski et al., 2003; Yukawa et al., 2007; Lv et al., 2011). The availability of genetic data for *C. glutamicum* led to the discovery of genetic clusters encoding aromatic degradation pathways with the aid of genomic bioinformatics. As *C. glutamicum* possesses catabolic pathways for the utilization of all major aromatic compounds present in biomass hydrolysates, it can be grown in the presence of lignin-derived aromatics (Shen et al., 2005; Sakai et al., 2007). Moreover, *C. glutamicum* is known to secrete L-lactate, succinate, and amino acids at higher concentrations as fermentation end products from glucose due to its robust glycolytic pathway (Wieschalka et al., 2012; Tsuge et al., 2019b). Because of these remarkable features, *C. glutamicum* was selected as a development target for investigation of microbial omnivory in bioprocessing.

Amongst the various lignocellulosic biomass available in United States, corn stover (the biomass residue left from harvest of the corn from *Zea mays*) represents both a cheap and an abundant biomass feedstock available for bioconversion. As found in other plant biomass, cellulose, hemicellulose, and lignin make up 70–90% of its biochemical constituents (Saha, 2003; Wang, 2007; Öhgren et al., 2007; Girio et al., 2010). Through mechanical, chemical or biochemical pretreatment, these biopolymers can be converted into the corresponding monomers, such as glucose, xylose, arabinose, and various aromatics (Chen et al., 2016). These monomers can in turn be used as carbon substrates in bioprocessing for conversion into fungible fuels and chemicals by a suitable biocatalyst like *C. glutamicum*.

**FIGURE 1:**
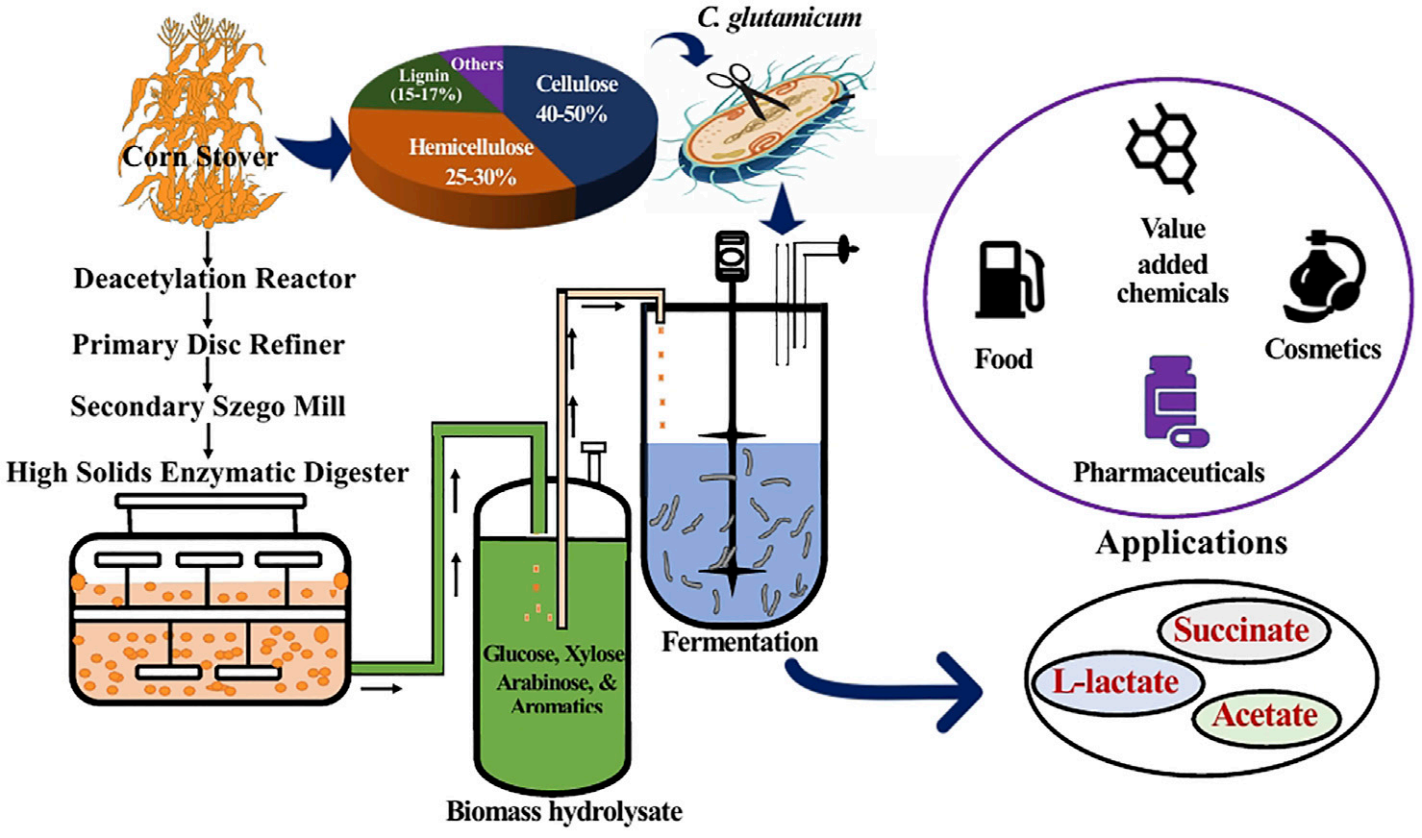
Schematic representation of the overall process employed in this study. Corn stover was subjected to deacetylation and mechanical refining (DMR) to obtain liquid biomass hydrolysate. The hydrolysate constituting glucose, xylose, arabinose, and aromatics was used as fermentation feedstock for *C. glutamicum* to produce mixed acids having various applications.

Mixed-acid fermentation products containing lactate, succinate, and acetate have gained significant attention due to their potential application as platform chemicals for manufacturing of biobased chemicals and materials. Succinate has been identified as one of the top 12 building block chemicals by the U.S. Department of Energy, with various applications in the pharmaceutical, food, and agricultural indutries (Zheng et al., 2012; Meng et al., 2016; Flores et al., 2020). L-lactate, one of the two lactate optical isomers, is mainly used in the production of a bio-based polymer poly-L-lactic acid (PLLA). Stereochemistry of lactate has a significant effect on the material properties of polylactic acid (PLA). The two common types of polylactic acid are poly-L-lactic acid (PLLA) and poly-D-lactic acid (PDLA). Both types of lactic acids are semicrystalline polymers. However, the PDLA obtained from the racemic mixtures is amorphous (Casalini et al., 2019). PLLA is used as a packaging material for fresh fruit containers, drinking cups, lamination films, and other items that can replace petroleum derived plastics. In the present study, by using *C. glutamicum* as a microbial host we demonstrate bioproduction of L-lactate, succinate, and acetate from corn stover biomass hydrolysates with simultaneous utilization of sugars and aromatics (Figure 1). As aromatics metabolic pathways are not well understood in *C. glutamicum,* ^13^C-fingerprinting studies have been used to throw more light on the aromatics metabolic pathway and thereby, aid future engineering efforts.

## Materials and Methods

### Chemicals and Reagents

All organic solvents, glucose, xylose, arabinose, aromatics, and other chemicals used in this study were purchased from Sigma-Aldrich (St. Louis, MO, United States). ^13^C labeled glucose was purchased from Cambridge Isotope Laboratories (Tewksbury, MA, United States). The biomass hydrolysate used in this study was obtained from NREL and used without any further processing, unless otherwise stated. In brief, the biomass hydrolysate used in this study was obtained by dilute alkali-based Deacetylation and Mechanical Refining (DMR) pretreatment and enzymatic hydrolysis technology developed at NREL (Chen et al., 2016).

### Bacterial Strains and Plasmids

*C. glutamicum* ATCC 13032 was used as the microbial host in this work (Kalinowski et al., 2003). *E. coli* TOP10 was used as the host for all plasmids constructed in this study. Genetic manipulations were carried out by following standard procedures (Sambrook and Russell, 2001). The plasmids pEKEx-xylAxc-xylBec and pVWE1araBAD provided by Dr. Wendisch (Bielefeld University) were used for the metabolic engineering of *C. glutamicum* to enable xylose and arabinose utilization (Schneider et al., 2010; Meiswinkel et al., 2013). All the plasmids and bacterial strains used in this study are listed in Table 1.

**TABLE 1:** Bacterial strains and plasmids used in this study.

Strain	Description	Source
*E. coli* Top 10	General cloning host	Stratagene, La Jolla, CA
*C. glutamicum*	Wild-type, ATCC 13032	ATCC
SSL03	*C. glutamicum* strain harboring pEKEx-xylAxc-xylBec	This study
SSL09	SSL03 harboring pVWE1araBAD	This study
Plasmid		
pEKEx-xylAxc-xylBec	pEKEx3 based recombinant plasmid harboring *xylA,* and *xylB*	Meiswinkel et al. (2013)
pVWE1araBAD	pVWEx1 based recombinant plasmid harboring *araB, araA* and *araD*	Schneider et al. (2010)

### Transformation

Transformation of *C. glutamicum* was conducted for construction of SSL03 and SSL09 strains as described previously (van der Rest et al., 1999; Eggeling and Bott, 2005) with minor modifications. Briefly, overnight cultures of *C. glutamicum* were inoculated into a 1L conical flask containing 100 ml of BHIS medium (brain-heart infusion medium containing 91 g/L sorbitol) and grown at 30°C until the optical density at 600 nm [OD_600_] reached between 0.9 and 1.0. The cells were washed twice with ice-cold 10% (v/v) glycerol. Finally, the cells were resuspended in 1 ml of 10% (v/v) glycerol. For each transformation, 100 μL of the cells was mixed with at least 100 ng of DNA. Electroporation was then performed in a 0.2 cm electroporation cuvette and electroporation was done with a gene pulser system (Bio-Rad Laboratories, Richmond, CA), operated at 25 uF, 200 Ω and 2.5 kV, 5 ms. The electroporated cells were immediately transferred to 1 ml of fresh BHIS medium and recovered at 30°C for 2 h. Subsequently, the cultures were then plated onto a BHIS agar plate containing appropriate antibiotics. Recombinant colonies usually appeared after 24 h of plating. Single colonies were propagated on a fresh brain heart infusion (BHI) liquid medium containing appropriate antibiotics to serve as inoculum for conducting fermentation experiments.

### Media and Culture Conditions

*E. coli* strains were cultivated in LB medium supplemented with kanamycin (50 μg/ml), and/or spectinomycin (100 μg/ml) as required at 37°C in a shaker at 200 rpm or on solidified LB plates. *C. glutamicum* strains were cultured in BHI medium or BTM2 medium (modified from BT medium) as required (Kawaguchi et al., 2006). For L-lactate production, the cells were grown in BTM2 medium. The BTM2 medium composition is as follows (per liter): (NH_4_)_2_SO_4_ 7 g, KH_2_PO_4_ 0.5 g, K_2_HPO_4_ 0.5 g, MgSO_4_.7H_2_O 0.5 g, urea 2 g, NaHCO_3_ 8.4 g, 1 × vitamin solution, and 1 × trace metal solution. The 1,000x trace metal solution (per liter) contained FeSO_4_.7H_2_O 6 g, MnSO_4_.H_2_O 10 g, ZnSO_4_.7H_2_O 0.56 g, CuSO_4_.5H_2_O 0.2 g, and 1,000x vitamin solution (per liter) contained biotin 4 g and thiamine 4 g. In addition, the BTM2 medium was supplemented with 2% MOPS to provide additional buffering capacity during L-lactate production (Rouabhia et al., 2014). BHI agar plates were supplemented with kanamycin (50 μg/ml), and spectinomycin (200 μg/ml) as required to obtain colonies of the engineered strains at 30°C.

Single colonies were inoculated into 5 ml of BHI medium and cultured overnight in 50 ml culture tubes at 30°C and 250 rpm. For the fermentation experiments, 1% of the overnight seed culture was inoculated into BTM2 containing 4% glucose or aromatics (benzoic acid, cinnamic acid, vanillic acid, and p-coumaric acid) at varying concentrations and grown for 5 days at 30°C and 250 rpm.

### Analytical Methods

Growth of *C. glutamicum* was monitored by measuring optical density of cultures at 600 nm with an Infinite 200 Pro plate reader (Tecan, Männedorf, Switzerland). Estimation of glucose, xylose, arabinose, acetate, L-lactate, and succinate concentration in culture broths were analyzed using HPLC. The supernatants were collected, filtered, and injected onto an ion exchange HPLC column at 70^o^C under an isocratic condition with a flow rate of 0.5 ml/min using 0.005 M sulfuric acid in water as solvent. Compounds were monitored by a refractive index detector in positive mode (kept at 40°C) and their concentrations were calculated by integration of peak areas and comparison to a calibration curve prepared from pure standards. Furthermore, enzyme based D (-)/L (+) lactic acid detection kit (R-biopharm) was used to verify the optical purity of L-lactate.

For aromatics analysis, supernatant liquid obtained from above was analyzed by an Agilent Technologies 1,260 Infinity II HPLC system equipped with an Agilent Eclipse Plus Phenyl-Hexyl column (250 mm length, 4.6 mm diameter, 5 µm particle size). The mobile phase gradient profile was as follows: 30% B (0 min; 0.5 ml/min), 80% B (12 min; 0.5 ml/min), 100% B (12.1 min; 0.5 ml/min), 100% B (12.6 min; 1 ml/min), 30% B (12.8 min; 1 ml/min), 30% B (15.6 min; 1 ml/min), where solvent A was 10 mM ammonium acetate in water and solvent B was 10 mM ammonium acetate in 90% acetonitrile. Sample injection volumes of 5 µL and a column temperature of 50°C were used. Prior to analysis, samples were filtered through 0.45 µm centrifuge filters. Compounds were monitored by a UV detector and their concentrations were calculated by integration of peak areas and comparison to a calibration curve prepared from pure standards.

### Fermentation Culture Conditions

Batch fermentation experiments were conducted in 1 L bioreactors with 500 ml working volume (Bioflo 115, New Brunswick Scientific Inc., Enfield, CT, United States). *C. glutamicum* was grown in BHI broth until the OD_600_ value reached ∼3 and was used as inoculum. Batch fermentations were conducted by the addition of 1% (v/v) inoculum to the fermenter containing 500 ml of BTM2 medium with 40 g/L of glucose. pH was maintained at seven by adding 25% Ca(OH)_2_. Fed-batch fermentations experiment were conducted by the addition of glucose at 72 h. All other fermentation conditions were identical to the batch fermentation experiments. Aerobic fermentation was conducted by sparging the cultures with air at 1 vvm and by continuously stirring the culture at 300 rpm. Anaerobic conditions were maintained by growing the cells without sparging air or oxygen. It can be verified from Supplementary Figure S2 that growing the cultures without sparging air or oxygen allowed the dissolved oxygen (DO) levels to be maintained at 0% except for the initial 10–12 h period.

A batch fermentation experiment with DMR hydrolysate was conducted with *C. glutamicum* SSL09 in a 1-L fermenter with 500 ml working volume (Bioflo 115, New Brunswick Scientific Inc., Enfield, CT, United States). The other fermentation conditions were identical to the batch fermentation experiment, with pH maintained at seven by adding 25% Ca(OH)_2_. Similarly, a batch fermentation experiment was conducted with BTM2 medium supplemented with pure sugars i.e. 40 g/L glucose, 20 g/L xylose, and 10 g/L arabinose supplemented with kanamycin (50 μg/ml), and spectinomycin (200 μg/ml). The rest other fermentation conditions were identical to the batch fermentation experiment conducted above.

### ^13^C Labeling Studies to Decipher Carbon Contribution by Aromatics

As a first step, *C. glutamicum* seed cultures were grown in BHI medium as described previously. Cells were centrifuged and washed with minimal medium to avoid any carryover of carbon substrates and nutrients from BHI medium. For the labeling study, 50 µL of washed cells were inoculated into 5 ml of BTM2 medium containing 5 g/L [U-^13^C] glucose and/or different aromatics at 20 mM concentration. Likewise, 2.5 g/L [U-^13^C] xylose with 9.25 g/L BHI in BTM2 minimal medium was used to investigate the presence of catabolic pathways for metabolizing xylose in *C. glutamicum*. Bacterial cells were harvested at the end of third day and the biomass was hydrolyzed to obtain proteinogenic amino acids. The amino acids were derivatized with TBDMS (N-(tert-butyldimethylsilyl)-N-methyl-trifluoroacetamide, Sigma-Aldrich) by following previously reported protocols (You et al., 2012; Varman et al., 2014). The derivatized amino acids were analyzed for their mass isotopomer abundance by GC-MS, as described elsewhere (Xiao et al., 2012; You et al., 2012). The m/z ion [M-57]^+^ that corresponds to the entire amino acid was used to calculate the mass fraction of labeled and unlabeled amino acids. m0, m1,…., mn represents mass distribution vector, where m0 is mass fraction of amino acids with zero labeled carbon, m1 is mass fraction of amino acids with one labeled carbon, and so on. The m/z of [M-15]^+^ was used for leucine and isoleucine since their [M-57]^+^ overlaps with other mass peaks (Wahl et al., 2004). The natural abundance of isotopes, including ^13^C (1.13%), ^18^O (0.20%), ^29^Si (4.70%), and ^30^Si (3.09%) contributes noise to the mass isotopomer spectrum. This background noise was rectified in the calculation of the mass distribution vector using a published algorithm and the detailed correction protocol can be found elsewhere (Tang et al., 2009). ^13^C fraction (^13^C carbons/total carbons) for amino acids were calculated using following formula (Xiao et al., 2013): ^13^C $\text{fraction}=1/n\overset{n}{\underset{k=0}{\sum }}{\text{k*m}}_{k}$ , where “n” is maximum number of carbons in the amino acid, m_k_ is mass fraction of amino acids with “k” number of labeled carbon.

## Results and Discussion

### Bioreactor Studies for Mixed Acid Production From Glucose

**FIGURE 2:**
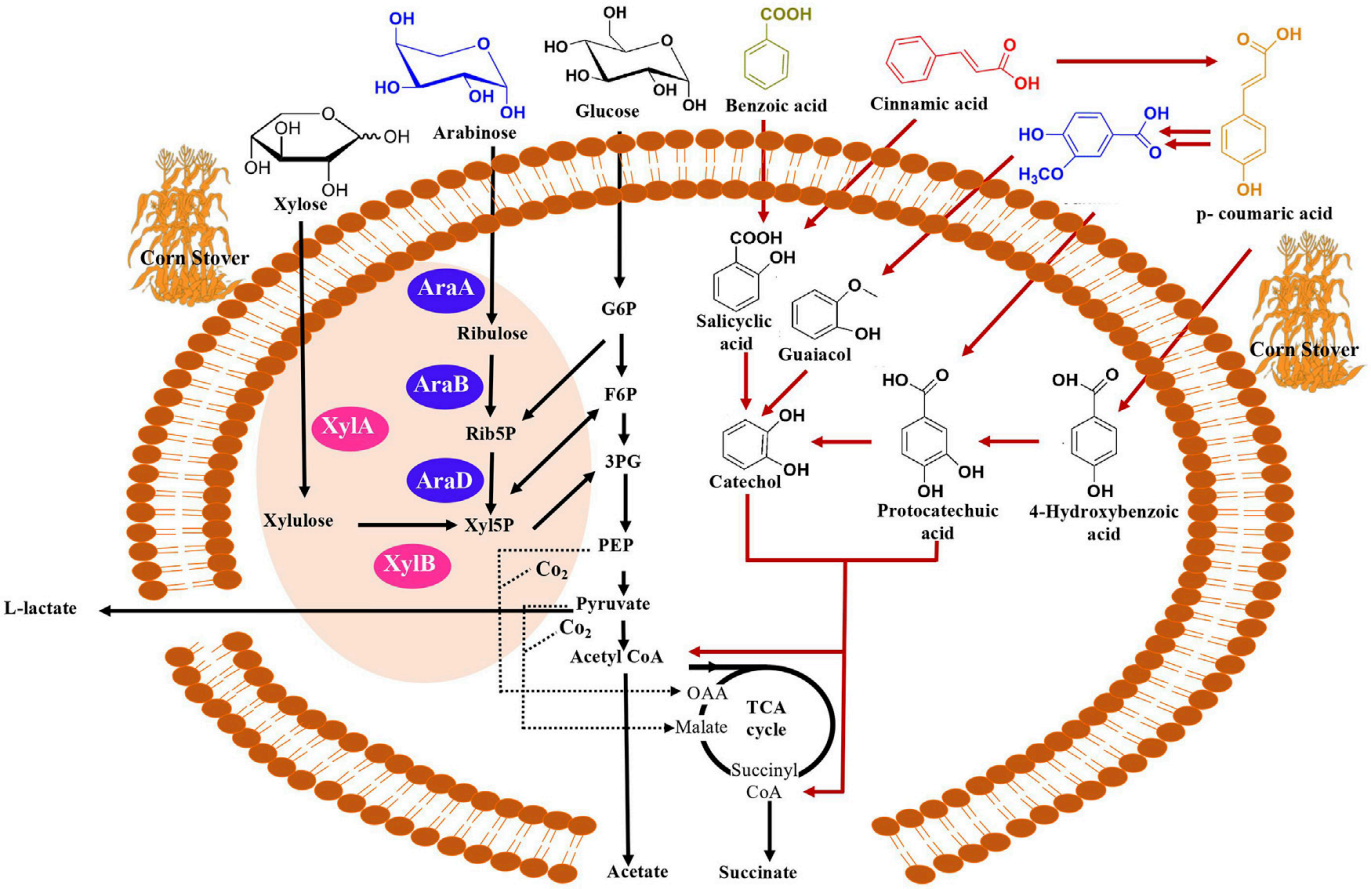
Overview of natural and engineered metabolic pathways for the production of mixed acids (succinate, acetate and lactate) from diverse carbon substrates (xylose, arabinose, glucose, benzoic acid, cinnamic acid, vanillic acid, p-coumaric acid) in *C. glutamicum.* Assimilation of xylose to be facilitated by XylA (xylose isomerase) and XylB (xylulose kinase) denoted by pink in the figure and assimilation of arabinose facilitated by AraA (arabinose isomerase), AraB (ribolokinase) and AraD (ribulose 5-phosphate 4-epimerase) denoted by purple. Abbreviations in arabinose and xylose assimilation Rib5P (ribulose 5-phosphate), Xyl5P (D-xylulose 5-phosphate), G6P (glucose 6-phosphate), F6P (fructose 6-phosphate), 3PG (3-phosphoglycerate). Succinate is synthesized through the reductive branch of TCA cycle from pyruvate or PEP (phosphoenolpyruvate) under anaerobic conditions (Shirai et al., 2007; Thakker et al., 2012). Acetate is synthesized from acetyl-CoA and L-lactate is synthesized via pyruvate. Aromatic catabolic pathway represented by red arrows. Anaplerotic pathways denoted by dotted lines.

**FIGURE 3:**
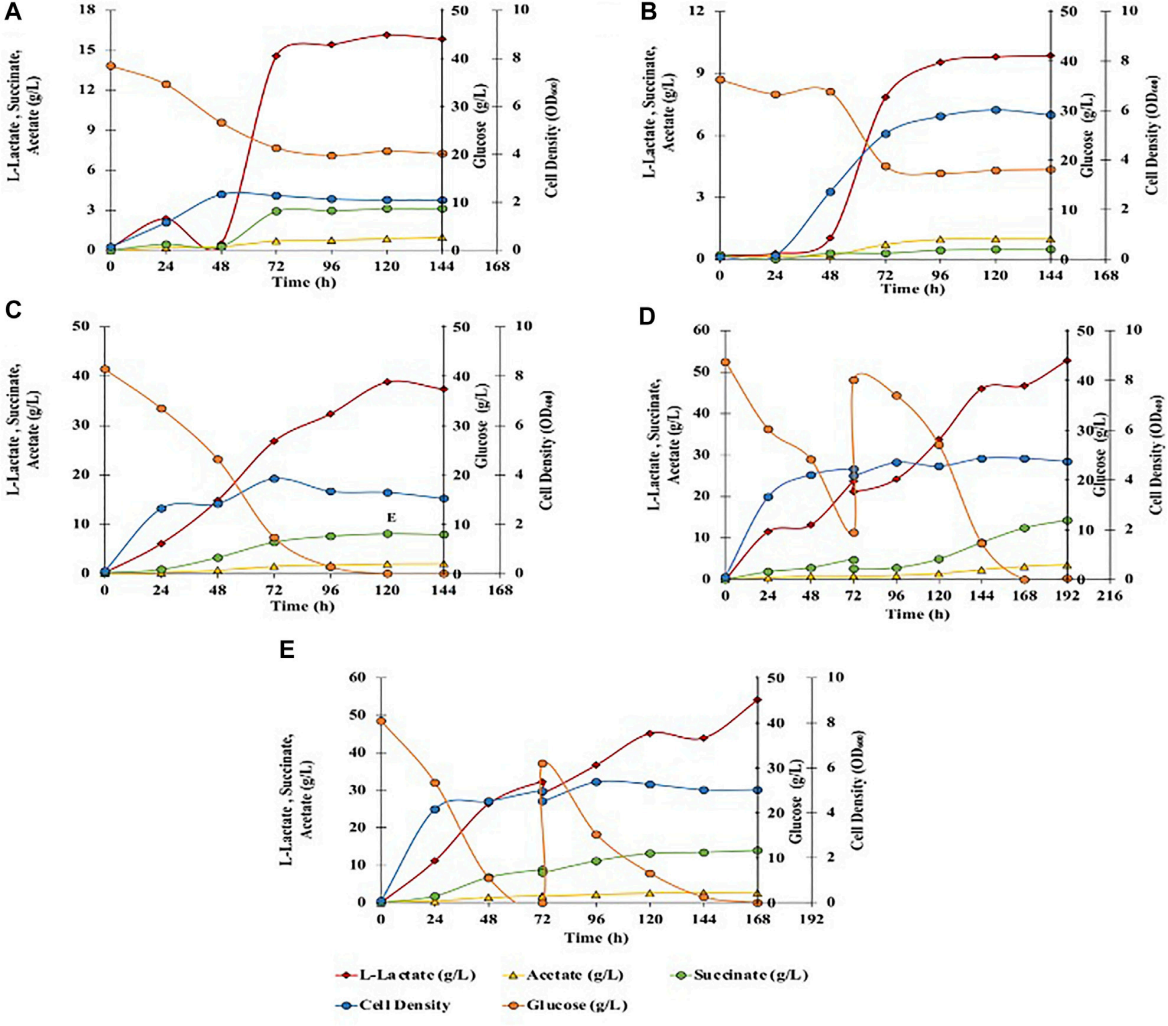
Effect of different fermentation cultivation parameters on mixed-acids production by *C. glutamicum.* Data is shown for single fed batch fermentation. **(A)** Anaerobic, batch, no pH control, BTM2 supplemented with glucose; **(B)** Aerobic, batch, no pH control, BTM2 supplemented with glucose; **(C)** Anaerobic, batch, pH 7, BTM2 supplemented with glucose; **(D)** Anaerobic, fed-batch: 40 g/L (0 h) + 30 g/L (72 h), pH 7, BTM2; **(E)** Anaerobic with initial aeration for 16 h, fed-batch: 40 g/L (0 h) + 30 g/L (72 h), pH 7, BTM2.

Glucose is the primary carbon substrate for *C. glutamicum,* which is assimilated through central metabolism and leads to the formation of various fermentation end products such as L-lactate, succinate, and acetate (Inui et al., 2004; Tsuge et al., 2015). L-lactate is produced from pyruvate and succinate is produced in the TCA cycle, whereas acetate is formed via acetyl-CoA (Figure 2). Fermentations with 40 g/L of glucose in BTM2 medium were performed to evaluate the L-lactate fermentation ability of *C. glutamicum* under aerobic and anaerobic conditions. As shown in Figure 3A, after 120 h of fermentation, 16.2 g/L of L-lactate was produced under anaerobic conditions along with 0.9 g/L and 3.1 g/L of acetate and succinate, respectively. Aerobic fermentation produced 9.8 g/L of L-lactate at a yield of 0.265 g/g from glucose, in comparison to 0.42 g/g obtained in the anaerobic fermentation (Table 2 and Figure 3B. Under aerobic conditions, pyruvate (precursor for L-lactate) produced in the glycolytic pathway is further metabolized in the citric acid cycle, thereby reducing the availability of pyruvate for lactate production. In the absence of oxygen, pyruvate is instantaneously converted to L-lactate (Okino et al., 2008; Chai et al., 2016). As a result, higher yields and titers of L-lactate were observed under anaerobic conditions.

**TABLE 2:** Fermentation kinetics of different cultivation strategies employed for production of mixed acids.

Batch	Initial glucose (g/L)	Initial xylose (g/L)	Initial arabinose (g/L)	% Glucose consumption	% Xylose consumption	% Arabinose consumption	Mixed acids titer		
							Acetate (g/L)	L- lactate (g/L)	Succinate (g/L)
I	36.21	0	0	50	−	−	1	9.6	0.5
II	38.47	0	0	46	−	−	0.9	16.2	3.1
III	41.45	0	0	100	−	−	2.1	38.6	8.1
IV	43.79	0	0	100	−	−	3.6	52.8	14.3
V	40.45	0	0	100	−	−	2.7	54.1	14
VI	37.30	18.15	8.05	100	17%	60%	2	31.5	8.1
VII	45.13	29.91	0	100	15%	−	3.2	47.9	8.9

Where BTM2 is modified BTM medium supplemented with sugar/s.

I- Aerobic, batch, BTM2 supplemented with glucose, no pH control, 120 h.

II- Anaerobic, batch, BTM2 supplemented with glucose, no pH control, 120 h.

III- Anaerobic, batch, BTM2 supplemented with glucose, pH 7, 120 h.

IV- Anaerobic, fed-batch: 40 g/L (0 h) + 30 g/L (72 h), BTM2 supplemented with glucose, pH 7, 192 h.

V- Anaerobic with initial aeration for 16 h, Fed-batch: 40 g/L (0 h) + 30 g/L (72 h), BTM2 supplemented with glucose, pH 7, 168 h.

VI- Anaerobic, batch; BTM2 supplemented with glucose, xylose and arabinose, pH 7, 168 h, SSL09.

VII- Anaerobic, batch; DMR hydrolysate, pH 7, 168 h, SSL09.

Energy metabolism in *C. glutamicum* gets impaired when the pH is maintained below six or above 8.5 owing to the non-generation of proton motive force (pmf) (Follmann et al., 2009). Therefore, it is crucial to maintain an optimal pH to maximize flux through the energy metabolism and to increase the product titer. Therefore, pH dependence of product formation was investigated at identical substrate loading to evaluate L-lactate production under chemo statically controlled neutral pH (pH 7). Ca(OH)_2_ has been effectively used as a low cost neutralizing agent in the production of L-lactate (Zhu et al., 2007; Nakano et al., 2012; Liu et al., 2014). Downstream processing for the recovery of L-lactate from calcium precipitate is also established, and generally performed with the addition of sulfuric acid. Therefore, pH was maintained with the addition of 25% Ca(OH)_2._ As shown in Figure 3C, 38.6 g/L of L-lactate was produced after 120 h with an increase in L-lactate yield to 0.932 g/g glucose. In addition, acetate and succinate were also produced in small amounts as byproducts at a titer of 2 g/L and 8.1 g/L, respectively. Downstream seperations of mixed acids can be successfully performed using the emulsion liquid membrane and adsorbent resins method as shown by Lee and Kim (2011) and Nam et al. (2012). Acetate and succinate along with L-lactate have extensive industrial applications and present work investigates *C. glutamicum’s* ability to produce organic acids from lignocellulosic substrates.

After establishing batch culture setup, further work focused on setting up a fed batch culture to check if fed batch culture will lead to higher mixed acid yields. A fed-batch fermentation was carried out with addition of 40 ml of 400 g/L glucose stock solution to the fermentation broth at 72 h, and fermentation was continued for another 120 h. In the end of the fermentation time course, 52.8 g/L of L-lactate (0.71 g/g glucose) was produced with 3.1 g/L of acetate and 12.4 g/L of succinate (Figure 3D). To further enhance the L-lactate production, fermentation was performed in high density cultures. Towards this end, aeration was performed for initial 16 h to enhance the cell growth at the beginning of the fermentation and to accumulate microbial biomass. Aeration was ceased and 40 ml of 400 g/L glucose was added at 72 h of fermentation. Under these conditions, 54.1 g/L of L-lactate was produced with 2.7 g/L of acetate and 14 g/L of succinate with corresponding yield of L-lactate 0.758 g/g, and productivity of 0.322 g/L/h (Figure 3E). No further improvements in L-lactate yield suggests the presence of pathway bottlenecks and can be resolved by established metabolic engineering approaches for lactate synthesis (Papagianni, 2012; Upadhyaya et al., 2014; Tsuge et al., 2015), which is not the primary focus of this study.

### Utilization of Aromatic Compounds in *C. Glutamicum*

**FIGURE 4:**
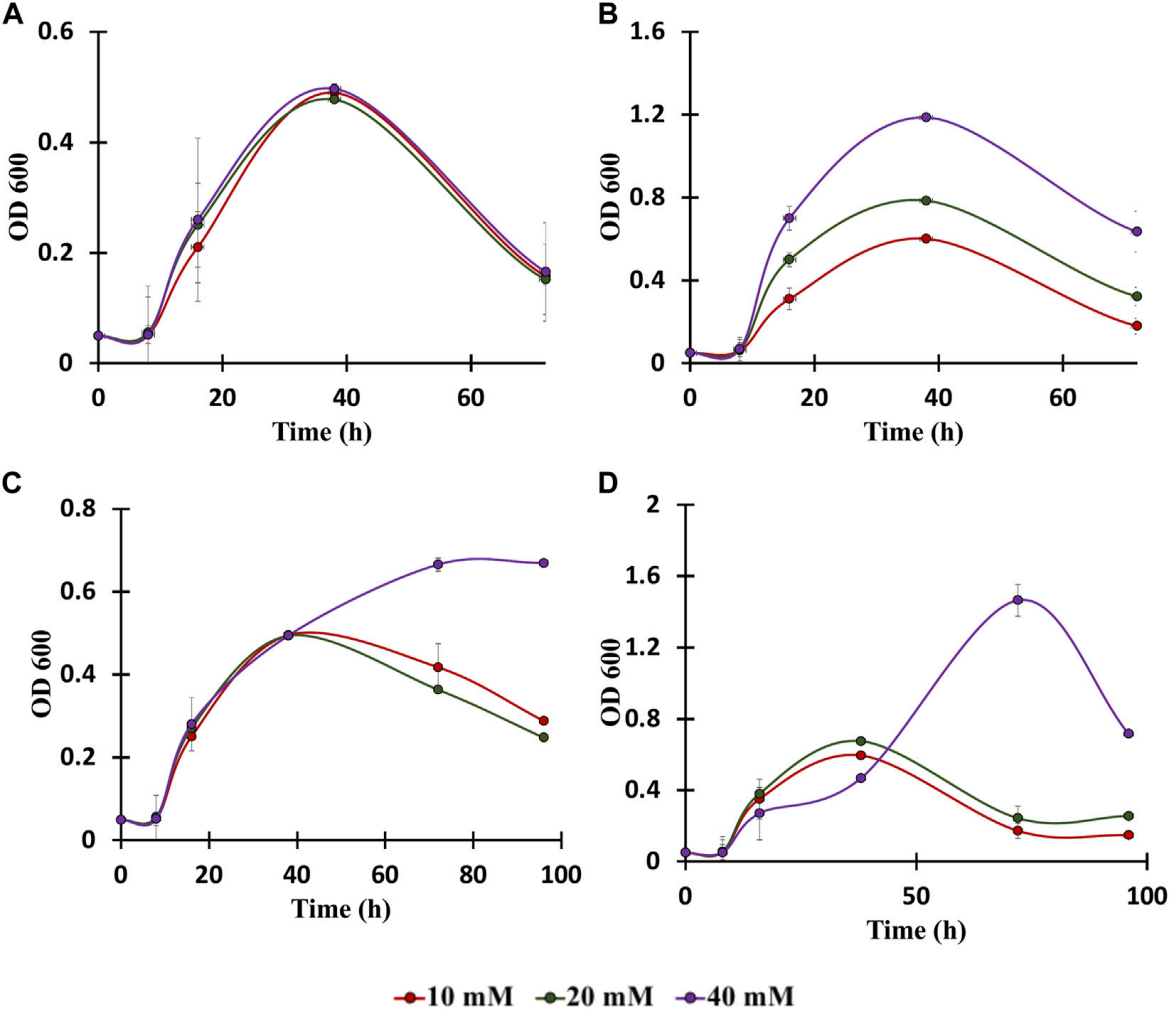
Growth assays of *C. glutamicum* in: **(A)** vanillic acid (10, 20, 40 mM), **(B)** Benzoic acid (10, 20, 40 mM), **(C)** Cinnamic acid (10, 20, 40 mM), and **(D)** p-coumaric acid (10, 20, 40 mM). The experiments were performed in biological triplicates. Data represents mean ± SD, *n* = 3.

To date, numerous advances have been made for efficient bioconversion of monomers derived from cellulosic and hemicellulosic biomass components (Tsuge et al., 2019a). *C. glutamicum* can play a major role in bioconversion of lignin-derived aromatics since it is highly tolerant to the presence of aromatics and it can grow by utilizing aromatics as the only carbon substrate (Figure 2) (Shen et al., 2012). In this context, genomic and proteomic analysis confirm the presence of genes and proteins for the utilization of aromatics through various peripheral pathways as shown in Figure 2 (Shen et al., 2012). Although it is known that the aromatic metabolic pathways are present, growth of *C. glutamicum* in the presence of aromatics has not being well characterized and the upper limit of aromatics concentration at which *C. glutamicum* still grows is unknown (Ding et al., 2015; Kallscheuer et al., 2016). Therefore, to further understand the limits of aromatics utilization, *C. glutamicum* strains were cultured in BTM2 medium containing various lignin derived aromatics (vanillic acid, benzoic acid, cinnamic acid and p-coumaric acid), in varying concentrations (10 mM, 20 mM, and 40 mM). *C. glutamicum* showed two folds higher growth in the presence of benzoic acid and p-coumaric acid as substrates compared to vanillic acid and cinnamic acid (Figure 4). Cells showed growth up to 40 mM concentration for all the aromatics which indicates high tolerance as well as successful utilization of lignin derived aromatics by *C. glutamicum*. Previous studies on *C. glutamicum* have tested its growth at lower aromatics concentration of 5–10 mM (Ding et al., 2015; Kallscheuer et al., 2016), and therefore, the present findings further expand the aromatics concentration limit for *C. glutamicum*. This is important from the context of lignin valorization as biomass hydrolysates on an average contain higher (more than 10 mM) lignin derived aromatics (Linger et al., 2014; Rodriguez et al., 2017; Gomes and Rodrigues, 2019; Salvachúa et al., 2020). In comparison, other microbial hosts being studied for the bioconversion of aromatics such as *Pseudomonas putida* KT2440, *P. putida* mt-2, *Rhodococcus jostii* RHA1, and *Sphingobium* sp. SYK-6 have been tested at aromatic concentrations less than 10 mM (Chen et al., 2012; Varman et al., 2016; Ravi et al., 2017; Kohlstedt et al., 2018; Almqvist et al., 2021; Spence et al., 2021). This remarkable ability of *C. glutamicum* to naturally resist as well as assimilate high concentrations of aromatics makes it an ideal choice for the bioconversion of multiple substrates present in biomass hydrolysates.

### ^13^C-Fingerprinting to Decipher Pathways for the Utilization of Lignin-Derived Aromatics

**FIGURE 5:**
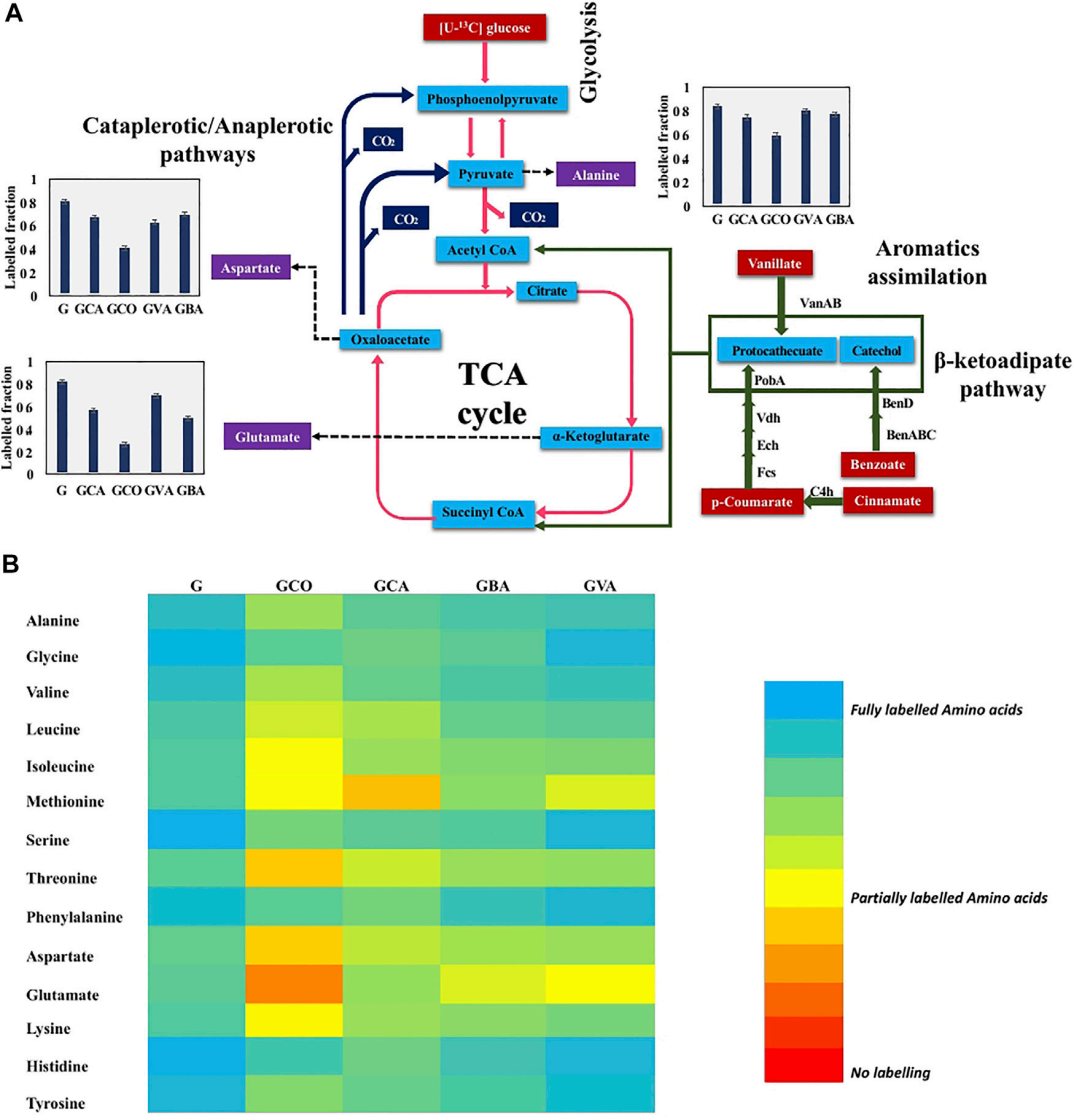
^13^C-fingerprinting study for tracing aromatic assimilation. Fully labeled [U-^13^C] Glucose (5 g/L) in BTM 2 medium was used in culturing *C. glutamicum* strain along with 20 mM concentration of aromatics to study aromatic preference in *C. glutamicum* strain. **(A)** Lignin derived aromatics (p-coumaric acid, cinnamic acid, vanillic acid, and benzoic acid) assimilation determination by tracing reduction in labeled fraction in key amino acids (alanine, glutamic acid, and aspartic acid). Cinnamate to p-coumarate catalyzed by C4h (cinnamate 4-hydroxylase), p-coumarate to protocathecuate catalyzed by Fcs (feruloyl-CoA synthetase), Ech (Enoyl-CoA hydratase), Vdh (vanillin dehydrogenase), and PobA (p-hydroxybenzoate hydroxylase), vanillate to protocathecuate catalyzed by VanAB (vanillate monooxygenase), and benzoate to catechol catalyzed by BenABC (benzoate 1,2-dioxygenase), BenD (1,6-dihydroxycyclohexa-2,4-diene-1-carboxylate dehydrogenase). Labeled fraction in aspartate, alanine, and glutamate is shown in charts where G: [U-^13^C] Glucose (5 g/L) in BTM 2 medium batch; GCA: [U-^13^C] Glucose (5 g/L) + 20 mM cinnamic acid in BTM 2 medium batch; GCO: [U-^13^C] Glucose (5 g/L) + 20 mM p-coumaric acid in BTM 2 medium batch; GVA: [U-^13^C] Glucose (5 g/L) + 20 mM vanillic acid in BTM 2 medium batch; GBA: [U-^13^C] Glucose (5 g/L) + 20 mM benzoic acid in BTM 2 medium batch. The experiments were performed in biological triplicates. Data represents mean ± SD, *n* = 3. **(B)** Heatmap depicting variation in labeled fraction in different amino acids where Glucose only: [U-^13^C] Glucose (5 g/L) in BTM 2 medium batch; GCA: [U-^13^C] Glucose (5 g/L) + 20 mM cinnamic acid in BTM 2 medium batch; GCO: [U-^13^C] Glucose (5 g/L) + 20 mM p-coumaric acid in BTM 2 medium batch; GVA: [U-^13^C] Glucose (5 g/L) + 20 mM vanillic acid in BTM 2 medium batch; GBA: [U-^13^C] Glucose (5 g/L) + 20 mM benzoic acid in BTM 2 medium batch.

To further elucidate the aromatics metabolic pathways, ^13^C-fingerprinting was performed. Specifically, [U-^13^C] glucose was fed to *C. glutamicum* along with the following unlabeled aromatics: p-coumaric acid, cinnamic acid, benzoic acid, and cinnamic acid in four independent batch cultures. The ^13^C-labeling analysis revealed that in comparison to the cultures fed with only labeled glucose, the cultures that were fed with both labeled glucose and aromatics showed a reduction in the labeling fraction of the amino acids (Figure 5). Interestingly, a minor reduction in labeled fraction was observed for cultures that were fed with only [U-^13^C] glucose. This can be attributed to the unlabeled carbon that entered via CO_2_ through anaplerotic pathways (PEP → OAA, pyruvate→OAA) (Shirai et al., 2007). This in turn contributed to unlabeled carbon in amino acids derived from TCA cycle such as glutamate and aspartate. These results confirm that *C. glutamicum* is able to effectively metabolize the aromatics and incorporate the carbon from the aromatics into cell biomass components. Interestingly, amino acids synthesized from the TCA cycle showed the highest reduction in the labeled carbon fraction (Figure 5B). This finding confirms that the unlabeled carbon skeletons from aromatics entered the primary metabolism via succinyl-CoA and acetyl-CoA derived from the β-ketoadipate pathway, as shown in Figure 5A (Shen et al., 2012).

Ubiquitous reduction in labeled fraction was observed in all four aromatics used in the present study. Amongst the various aromatics that were tested, the highest reduction in carbon labelling was observed with p-coumaric acid followed by cinnamic acid, benzoic acid and least in vanillic acid (Figure 5B). Specifically, labeling of glutamic acid and aspartic acid significantly reduced by 67 and 48%, respectively. However, alanine labeling was reduced by only 24%, which is strong evidence that carbon is entering the TCA cycle without being converted into pyruvate directly. This also suggest that p-coumaric acid is the aromatic preferred by *C. glutamicum*, which agrees with the higher growth rate observed in p-coumaric acid fed cultures compared to the other aromatics (Figure 5D). Interestingly, the p-coumaric acid catabolic pathway involves a greater number of steps compared to the catabolic pathways of vanillic acid or benzoic acid. One potential explanation can be that *C. glutamicum* has highly active enzymes in the coumarate catabolic pathway, resulting in better uptake and breakdown of coumarate. Another possibility is that the coumarate catabolic pathway is upregulated under these conditions compared to the other aromatics. Furthermore, these results are congruent with the growth assay results reported in Figure 4, which showed highest growth in the presence of p-coumaric acid among all the other aromatics that were tested. These results can be leveraged for the further engineering of *C. glutamicum* as we aspire to increase the catabolism of all aromatics simultaneously for increased production of aromatics derived fuels and chemicals. The p-coumaric acid catabolic pathway is a three-step process leading to the formation of protocatechuate as an intermediate. Protocatechuate is also an intermediate for both vanillate and cinnamate catabolic pathways. In general, protocatechuate degradation occurs via three different pathways known as the ortho, meta, and para cleavage pathways. Meta and para cleavage pathways provide pyruvate as an intermediate prior to the carbon entering the TCA cycle. Alanine labelling in an organism can be used to confirm the presence of these pathways, since alanine is directly biosynthesized from pyruvate. From Figure 5, it can be observed that aspartate and glutamate had higher reduction in labelling as compared to alanine which demonstrates that carbon enters the TCA cycle without being diverted through pyruvate. Therefore, this work confirms that the ortho-cleavage protocatechuate pathway is active in *C. glutamicum*, in agreement with the previous genomic studies (Shen et al., 2012; Kallscheuer et al., 2016). Furthermore, the 10–15% reduction in labeling fraction of alanine indicate that cataplerotic pathways are also active in *C. glutamicum*.

*C. glutamicum* has long been used for the production of amino acids, including glutamic acid and lysine. The present study shows that glutamic acid is highly labeled compared to aspartic acid, both derived from TCA cycle intermediates. This trend was consistent in all of the labeling studies that were performed with the various aromatics. A likely explanation for this observation is the presence of anaplerotic pathways, through which labeled carbon from pyruvate and phosphoenolpyruvate enters oxaloacetate to produce aspartic acid (Varman et al., 2016). Also, as *C. glutamicum* is a natural producer of glutamic acid, the flux from α-ketoglutarate to glutamate may be more robust, thereby restricting the unlabeled carbon flow from α-ketoglutarate to oxaloacetate. Overall, the ^13^C analysis performed improves our understanding of aromatic catabolism in *C. glutamicum,* and provides strong evidence that aromatics present in lignocellulosic biomass hydrolysates can be used as carbon substrates for the bioproduction of L-lactate using this chassis.

### Mixed-Acid Production From Corn Stover Hydrolysate

In the above sections, we have established fermentation of mixed acids in the presence of glucose as well as gained a better understanding of the aromatics metabolism in *C. glutamicum.* As a next step conducting fermentation with actual biomass hydrolysates containing diverse substrates will establish *C. glutamicum* as a omnivorous host for bioproduction. However, several of the previous studies have reported that *C. glutamicum* lacks the ability to catabolize xylose. On the other hand, whole genome sequencing has confirmed the presence of *xylB* that encodes for xylulokinase, but its function was never studied (Kawaguchi et al., 2006). This led us to wonder if the other genes of the xylose pathway are present in *C. glutamicum* but are not yet known. Therefore, to investigate the presence of xylose pathway in *C. glutamicum* ^13^C isotopomer analysis was performed by feeding the cells with [U-^13^C] xylose. We observed that there was no change in MDV levels for cells grown with labeled xylose in comparison to cells that were grown without labeled xylose (Supplementary Figure S3). The absence of labelling conclusively proves that *C. glutamicum* lacks the ability to metabolize xylose under the conditions tested in the laboratory. Therefore, it was essential to impart *C. glutamicum* the ability to grown on pentose sugars. Towards this, the genes *xylA*, *xylB* of xylose pathway, and the genes *araA*, *araB*, *araD* of arabinose pathway were transformed into the *C. glutamicum* ATCC 13032 strain resulting into the strain SSL09 (Schneider et al., 2010; Meiswinkel et al., 2013). Also, to evaluate the growth of *C. glutamicum* in the presence of biomass hydrolysates, the wild-type strain was grown in the presence of varying volume ratios of the hydrolysate. Biomass hydrolysates contain chemicals like furfurals and hydroxymethylfurfurals (HMF) which can be inhibitory to growth of bacterial cells (Tsuge et al., 2014; Chen et al., 2016; Kuhn et al., 2020). However, DMR hydrolysate has been processed to remove these inhibitors (Chen et al., 2016). *C. glutamicum* was found to be tolerant to the presence of aromatics and other unknown inhibitory chemicals present in corn stover biomass hydrolysates (DMR) (Chen et al., 2016), growing well even in the presence of 80% hydrolysates (Supplementary Figure S1). However, the maximum growth rate was observed in the presence of 20% (vol/vol) of biomass hydrolysate, therefore this hydrolysate concentration was chosen for further experiments.

**FIGURE 6:**
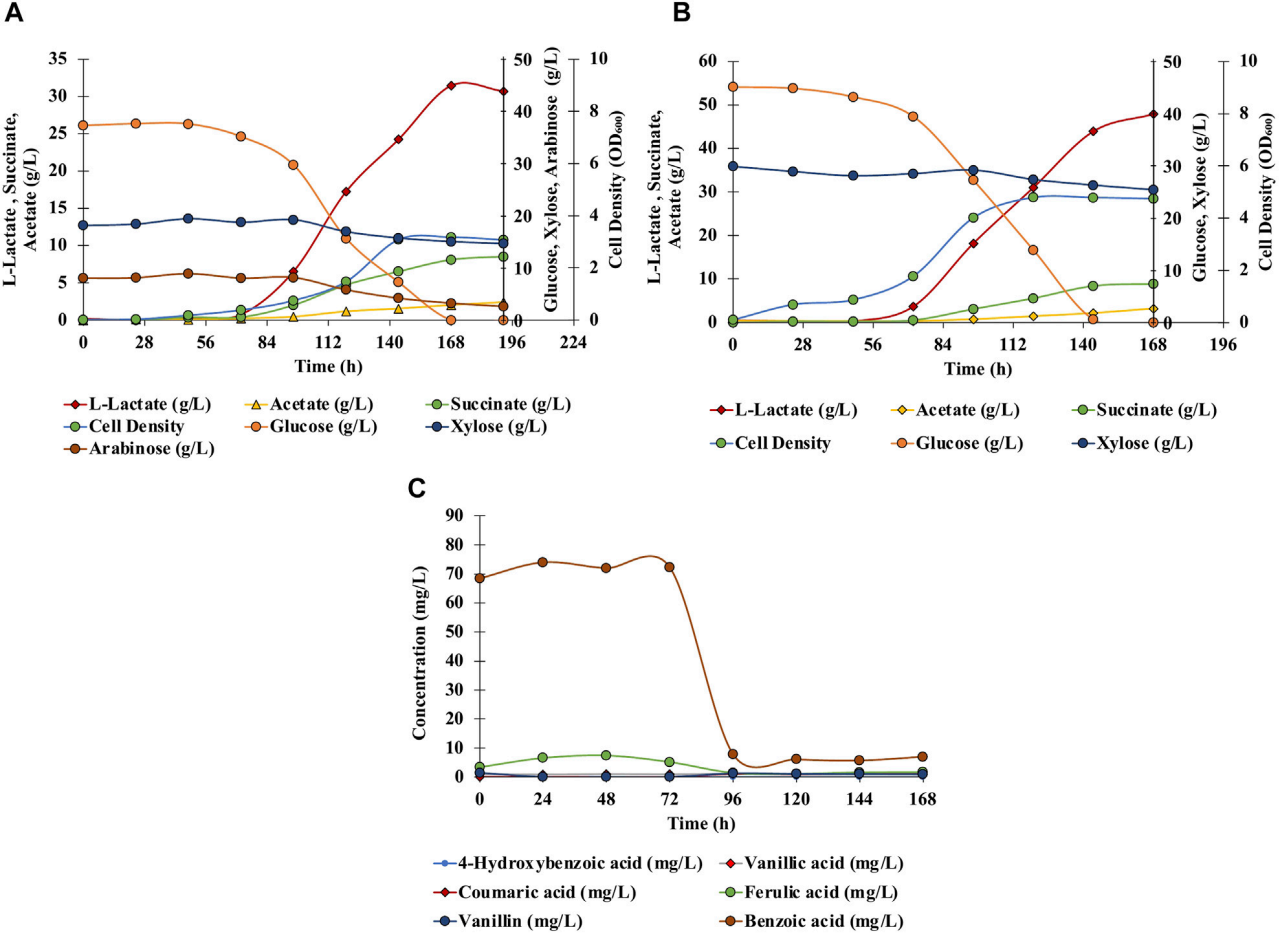
Effect of different fermentation cultivation parameters on mixed-acids production by *C. glutamicum.* Data is shown for single fed batch fermentation. **(A)** Anaerobic, batch, pH 7, BTM2 supplemented with glucose, xylose, and arabinose; **(B)** Anaerobic, batch, pH 7, DMR hydrolysate; **(C)** Aromatics utilization: anaerobic, batch, pH 7, DMR hydrolysate.

Anaerobic fermentation experiments were carried out using wild-type *C. glutamicum* and engineered SSL09 strains in BTM2 medium supplemented with glucose, xylose, and arabinose as carbon substrates. As seen in Figure 6A, complete utilization of glucose was observed in 168 h followed by utilization of arabinose and xylose. Delayed utilization of xylose and arabinose can be associated with the most common catabolite repression phenomenon observed in microorganisms in the presence of multiple carbon substrates (Dien et al., 2002; Abdel-Rahman et al., 2015; Flores et al., 2020). Although utilization of pentose sugars happened only after 90 h, other works have shown that through further engineering, *C. glutamicum* can be made to utilize both pentose and hexose sugars simultaneously (Sasaki et al., 2016). Lactate (31.5 g/L) along with acetate (2 g/L) and succinate (8.1 g/L) were produced at 168 h. Figure 6B shows the fermentation profile using DMR corn stover hydrolysate as a fermentation medium. Lactate 47.9 g/L along with acetate 3.2 g/L and succinate 8.9 g/L were produced with complete utilization of glucose in 144 h. Utilization of xylose (17%), arabinose (60%), glucose (100%) lead to production of acetate (2 g/L), L-lactate (31.5 g/L) and succinate (8.1 g/L) in SSL09 strain this shows active utilization of pentose and hexose sugar utilization for production of mixed acids. At the end of the fermentation time course, the yield of lactate from sugars available in DMR hydrolysate was 0.639 g/g of the total, and productivity was 0.285 g/L/h. Furthermore, *C. glutamicum* showed good growth in DMR hydrolysate and lead to reduction in aromatics levels (Figure 6C). In this fermentation trial, it was observed that the strain completely consumed the aromatics ferulic acid, vanillin, and benzoic acid which were present in DMR hydrolysate (Figure 6C). Although, we have demonstrated the production of mixed acids from 20% biomass hydrolysate, using high biomass concentration as feedstock is preferred from an industrial standpoint. However, the DMR hydrolysate used in this study contains very high total sugar concentration, 230 g/L (Chen et al., 2016). Growing *C. glutamicum* at high sugar concentrations would require adaptive laboratory evolution of the strain. Therefore, future works should focus on utilizing adaptive laboratory evolution to improve the tolerance of *C. glutamicum* at high sugar concentrations and further optimization of bioprocess conditions to grow the cells at very high hydrolysate levels. Overall, this work demonstrates the first application of *C. glutamicum* to utilize all the diverse carbon substrates present in lignocellulosic biomass for bioproduction of value-added chemicals.

## conclusion

The omnivorous strain *C. glutamicum* ATCC 13032 was investigated for production of mixed acids from sugars and aromatics present in lignocellulosic biomass. Fermentative processes in a variety of cultivation modes, including an aerobic cell growth and anaerobic fermentation phase, batch, and fed-batch mode, using BTM2 medium with different carbon substrates, and DMR corn stover hydrolysate were evaluated for production of mixed acids from pure sugars as well as biomass hydrolysate. In all cultivation modes, L-lactate was produced in significantly high titers with acetate and succinate as co-products. *C. glutamicum* exhibited a maximum productivity of 0.322 g/L/h towards L-lactate with 54.1 g/L when grown aerobically for the first 16 h followed by anaerobic fermentation. Also, utilization of pentose and hexose sugars as well as aromatics present in corn stover hydrolysate for mixed acid production was demonstrated. Furthermore, *C. glutamicum* showed high tolerance and utilization of different aromatics commonly present in lignocellulosic hydrolysate, including benzoic acid, cinnamic acid, vanillic acid, and p-coumaric acid. ^13^C-fingerprinting confirmed the roles of the ortho-cleavage pathway, anaplerotic pathway, and cataplerotic pathways for utilization of aromatics in the TCA cycle, with p-coumaric acid as the preferred substrate amongst aromatics studied. These findings suggest *C. glutamicum* is a versatile omnivorous host for valorization of lignocellulosic feedstocks.

## Data Availability

The raw data supporting the conclusions of this article will be made available by the authors, without undue reservation.
