# Corrigendum: *Corynebacterium glutamicum* as an efficient omnivorous microbial host for the bioconversion of lignocellulosic biomass

**DOI:** 10.3389/fbioe.2022.1116067

**Published:** 2022-12-20

**Authors:** Apurv Mhatre, Somnath Shinde, Amit Kumar Jha, Alberto Rodriguez, Zohal Wardak, Abigail Jansen, John M. Gladden, Anthe George, Ryan W. Davis, Arul M. Varman

**Affiliations:** ^1^ Chemical Engineering Program, School for Engineering of Matter, Transport, and Energy, Arizona State University, Tempe, AZ, United States; ^2^ Department of Bioresource and Environmental Security, Sandia National Laboratories, Livermore, CA, United States; ^3^ Department of Biomaterials and Biomanufacturing, Sandia National Laboratories, Livermore, CA, United States; ^4^ Joint BioEnergy Institute, Emeryville, CA, United States

**Keywords:** lignocellulosic biomass hydrolysate, ^13^C-fingerprinting, lignin-derived aromatics, mixed-acid fermentation, L-lactate

In the published article, there was an error in the caption of Figure 4 as published. The concentrations of vanillic acid, cinnamic acid, benzoic acid and coumaric acid (mM) displayed were denoted as 40, 80, 150 mM. However, the correct concentrations are 10, 20 and 40 mM. The corrected legend appears below.

**FIGURE 4:**
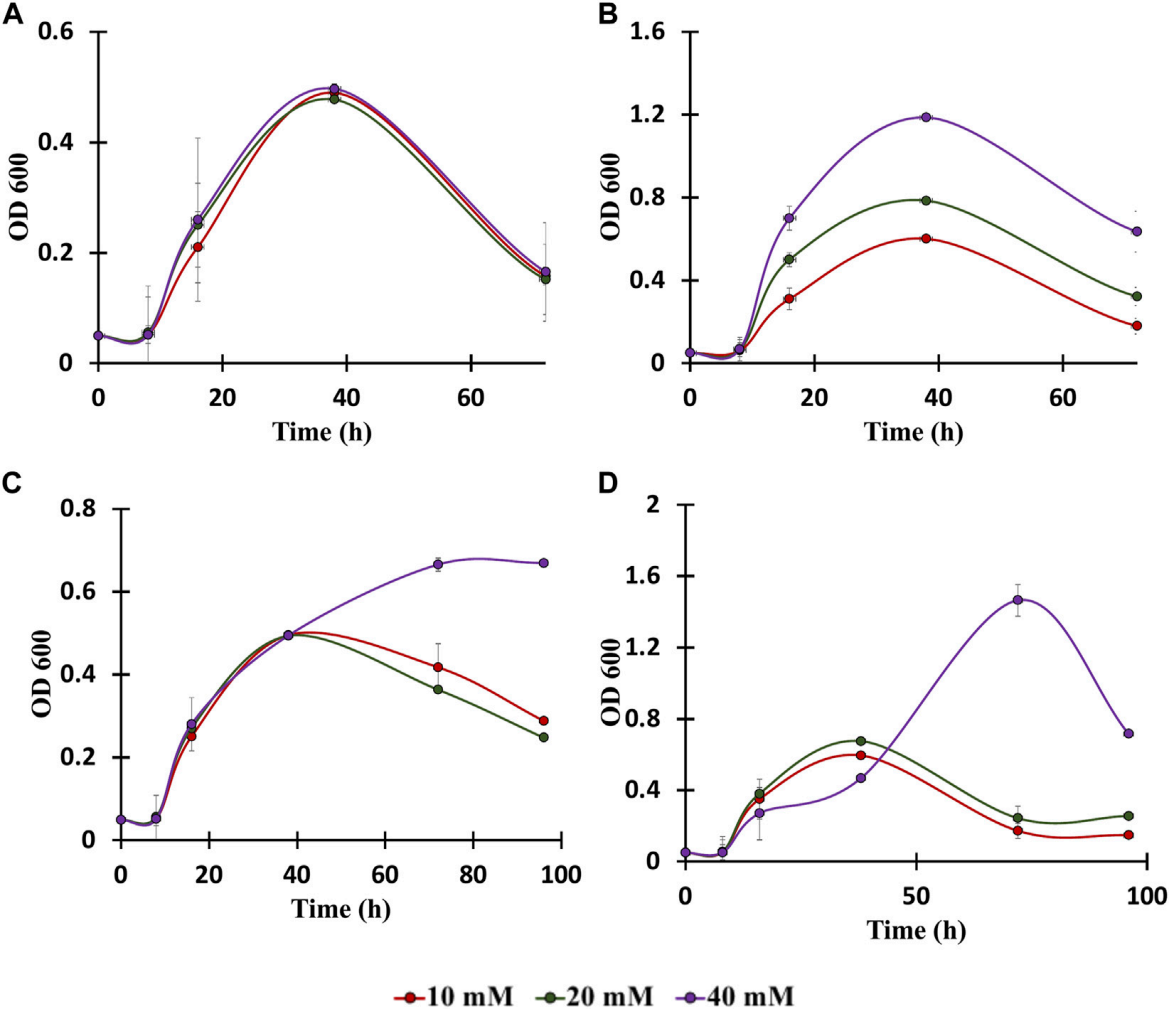
Growth assays of *C. glutamicum* in: **(A)** vanillic acid (10, 20, 40 mM), **(B)** Benzoic acid (10, 20, 40 mM), **(C)** Cinnamic acid (10, 20, 40 mM), and **(D)** p-coumaric acid (10, 20, 40 mM). The experiments were performed in biological triplicates. Data represents mean ± SD, *n* = 3.

The authors apologize for this error and state that this does not change the scientific conclusions of the article in any way. The original article has been updated.

