# Supplementary material for: Corynebacterium glutamicum as an Efficient Omnivorous Microbial Host for the Bioconversion of Lignocellulosic Biomass
Source: Front Bioeng Biotechnol. 2022 Apr 1;10:827386. doi: 10.3389/fbioe.2022.827386 (PMC9011048; doi:10.3389/fbioe.2022.827386)
Supplement: Supplementary file 1 [file DataSheet1.pdf]

Supplementary information

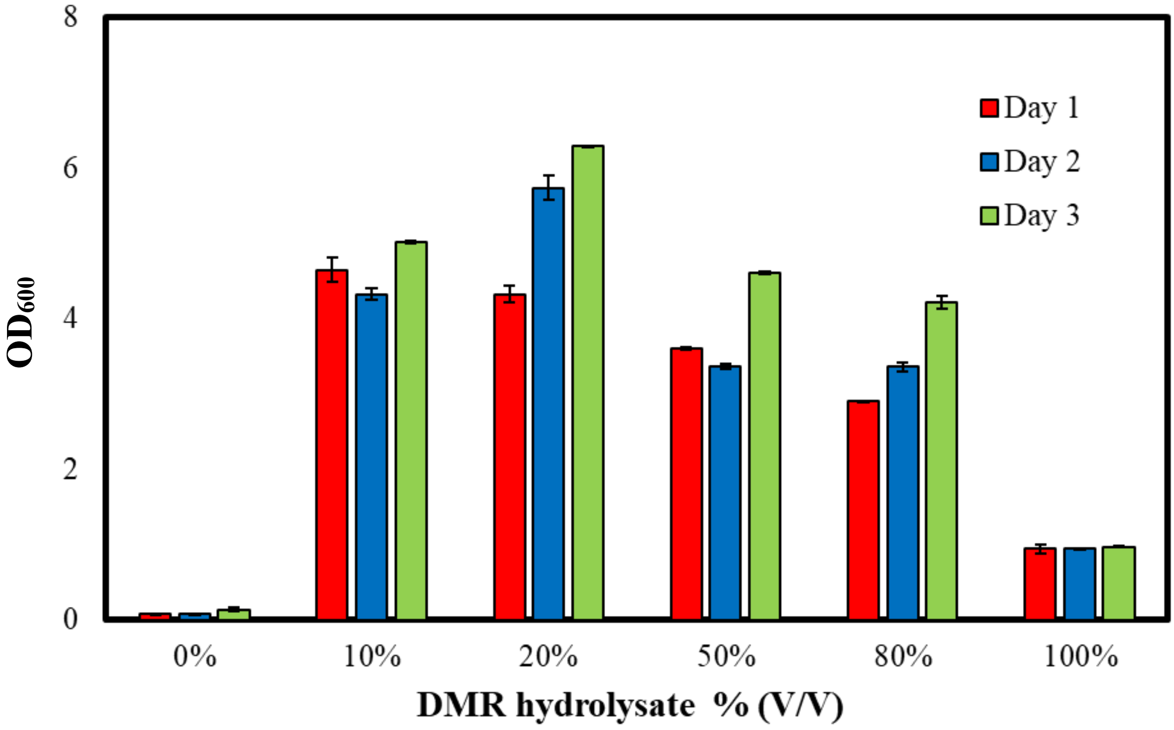

Figure S1: Effect of varying DMR hydrolysate concentrations on the growth (Data represents mean ± SD, n=3) of the *C. gluatmicum* strain ATCC 13032.

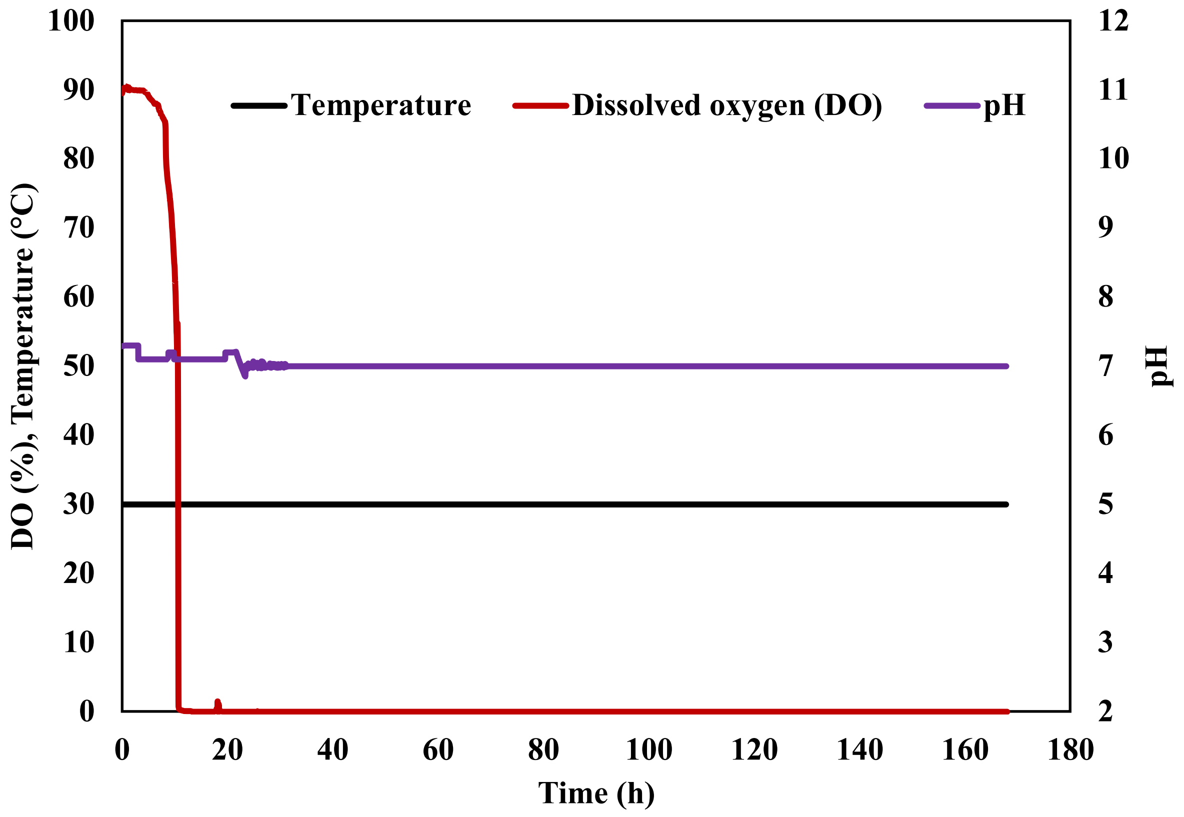

Figure S2: Variations in dissolved oxygen, temperature, and pH with time in a bioreactor during anaerobic fermentation.

A

B

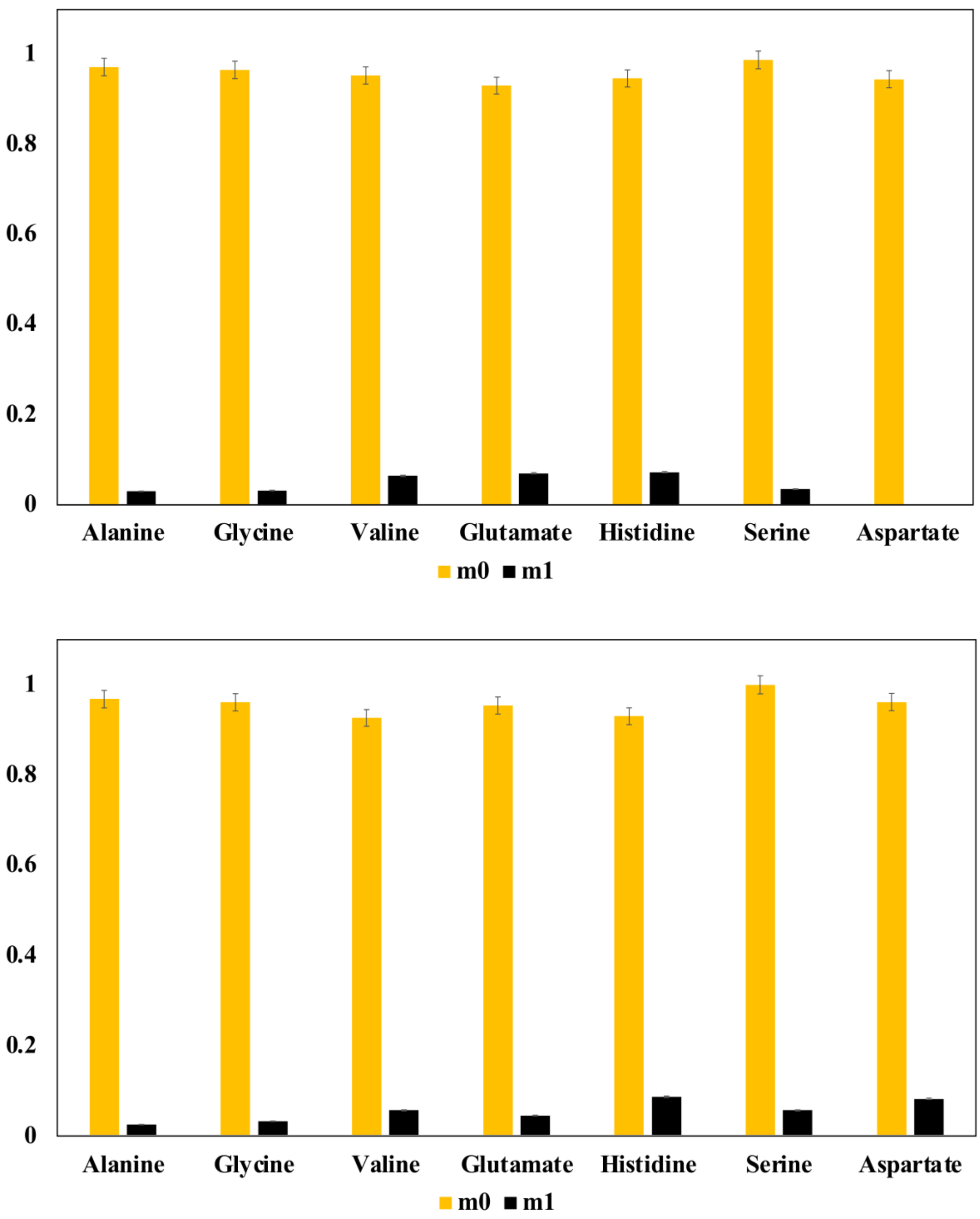

Figure S3: Mass distribution of amino acids for *C. glutamicum* grown in (A): [U-^{13}C] xylose in BTM2 media supplemented with BHI; B) BTM2 media supplemented with BHI but no extra sugars. Error bars indicate 2% technical error of instrument.
